# Genetic Predisposition to Pancreatic Cancer: A Systematic Review of Hereditary Syndromes and Familial Aggregation

**DOI:** 10.3390/cancers18060976

**Published:** 2026-03-18

**Authors:** Catalin Sergiu Baraian, Claudiu Stefan Turculet, Ionut Negoi

**Affiliations:** Carol Davila University of Medicine and Pharmacy Bucharest, Clinical Emergency Hospital of Bucharest, 014461 Bucharest, Romania; claudiu.turculet@umfcd.ro

**Keywords:** pancreatic cancer, screening, inherited predisposition, genetic syndromes, familial clustering, high-risk mutations, genetic counseling

## Abstract

Background: Pancreatic cancer is a highly lethal malignancy, with a 5-year survival rate of approximately 8%. Roughly 10% of cases occur in individuals with familial pancreatic cancer or identified high-risk germline mutations, including STK11, CDKN2A, BRCA1/2, MLH1, and MSH2. Aim: We aimed to evaluate the risk of pancreatic cancer associated with inherited genetic mutations and to characterize these genetic syndromes. Methods: A systematic search of the PubMed database up to 2024 identified 1500 articles, of which 90 met the criteria for inclusion in this review. Results: High-risk individuals were defined as those with at least a 10-fold increased risk, moderate risk as 5–10-fold and low risk as under 5-fold. High-risk individuals included those with Peutz–Jeghers syndrome (132–140-fold risk), hereditary pancreatitis (50–87-fold risk), Familial Atypical Multiple Mole Melanoma syndrome (up to 48-fold risk), hereditary breast and ovarian cancer with BRCA2 mutation (up to 22-fold risk), and familial pancreatic cancer with at least three affected relatives (up to 32-fold risk). Moderate-risk patients had BRCA1, MLH1, MSH2, MSH6, p53, and ATM mutations, as well as familial pancreatic cancer with 1–2 affected kindred. Low-risk patients had familial adenomatous polyposis. Conclusions: Identifying high-risk individuals is crucial for effective genetic counseling, testing, and potential screening programs to facilitate early diagnosis and improve outcomes. Future research should prioritize large prospective cohorts, screening programs, and the integration of emerging technologies, such as AI-assisted imaging.

## Simple Summary

Pancreatic cancer has a high mortality rate despite its low incidence. Of all cases, approximately 10% are linked to inherited genetic mutations or familial clusters. High-risk individuals are those with Peutz–Jeghers syndrome, hereditary pancreatitis, Familial Atypical Multiple Mole Melanoma syndrome, hereditary breast and ovarian cancer with BRCA2 mutation and clusters of familial pancreatic cancer with at least three affected kindreds. Early detection through targeted screening in these patients may improve the outcomes. Genetic counseling and multigene testing are essential for risk stratification and management.

## 1. Introduction

Pancreatic cancer, whether of exocrine or endocrine origin, has a worldwide incidence of 2.6%, which places it 13th among all types of cancers in 2022. However, regarding overall mortality, pancreatic cancer is the 6th cause of death among cancers, responsible for 4.8% of deaths [1]. The survival rate has improved over the last few decades, but it remains one of the cancers with the worst survivability. In the United Kingdom, the 1-year survival rate improved from 12.2% in 1995 to 26.3% in 2014, while the 5-year survival rate improved from 2.9% to 7.93% [2].

Among all pancreatic cancer patients, it is estimated that approximately 5–10% [3] are caused by specific mutations that appear in different genetic syndromes or belong to familial clusters. Despite the lack of data regarding the genetic mutations in familial clusters, it is known that the risk of developing pancreatic cancer is higher in high-penetrance genes such as BRCA1 and 2, STK11, LKB1, PALB2, PRSS1, SPINK1, and CDKN2A, and low-penetrance genes such as ABO and CFTR [4,5]. Given these data, we are currently witnessing a shift from traditional treatment (chemotherapy, radiotherapy, and surgery) to emerging approaches, with the introduction of gene therapy, albeit still experimental. Genetic counseling should be mandatory for such patients. It is important to note that starting with the 2021 NCCN Pancreatic Cancer Guidelines, it is recommended that all pancreatic cancer patients undergo germline mutation testing [6].

The cardinal features of hereditary pancreatic cancer are the age of diagnosis (early onset), a pattern of multiple cancers in family members, especially close relatives, possible distinguishing clinical and pathological features, and Mendelian transmission (mostly autosomal dominant or recessive) [7].

Early detection of pancreatic cancer, ideally as a precancerous lesion, offers the best hope of reducing mortality in pancreatic cancer. Patients with adenocarcinoma of less than 1 cm in diameter (T1a) have a survival rate of 80% at 5 years. Furthermore, many pancreatic cancers arise from premalignant lesions, such as Intraductal Papillary Mucinous Neoplasm (IPMNs), which are curable by surgery. Hence, screening for pancreatic cancer can save lives. However, since the incidence of this cancer is 8.5–12/100,000, it is not feasible to screen the general population. However, it is possible to screen high-risk patients [8].

## 2. Materials and Methods

### 2.1. Eligibility Criteria

This study is a systematic review conducted in accordance with PRISMA statement. The protocol was designed to identify, evaluate and synthesize all available evidence regarding genetic predisposition in pancreatic cancer.

A PRISMA checklist can be found in the Supplementary Materials [9].

A PICO strategy was devised as follows: P = individuals with hereditary genetic syndromes or familial aggregation with an increased risk of developing pancreatic cancer. I = evaluation of the risk of developing pancreatic cancer. C = compared with the general population. O = early detection of pancreatic cancer in individuals.

There were no exclusion criteria, and all screened studies were included according to the search strategy. The current review was registered with OSF on 4 September 2025 under DOI 10.17605/OSF.IO/VGMJZ.

### 2.2. Information Sources and Search Strategy

A comprehensive search strategy was developed following the PRISMA guidelines, and the search was performed on PubMed for all articles up to the end of 2024. The following search parameter was used: (((Pancrea*) AND ((Neoplasm*) OR (Tumor*) OR (Mass*) OR (Growth))) OR (“Pancreatic Neoplasms”[Mesh])) AND (((Genetic*) OR (Inherited) OR (Predisposition*) OR (Hereditary) OR (Familia*)) AND ((Syndrom*) OR (“Syndrome”[Mesh]))) AND (Screening). Subsequently, studies that followed a screening strategy were excluded if there were no data regarding the risk of developing pancreatic cancer in high-risk individuals.

PubMed was selected as the main database for this review because it is accessible and reliable library for medical literature. Given the focus of the current project, we anticipated that relevant clinical data would be found in this database. Moreover, reference list of the included studies was cross-referenced to ensure that all relevant studies would be included.

### 2.3. Selection and Data Collection Process

All articles were screened for eligibility by a single researcher in two steps. First, the titles and abstracts were screened to exclude irrelevant articles. Second, full texts were screened to determine the eligibility of each article. A few articles were translated using Google Translate (mostly from Chinese or German). A Generative AI Tool (ChatGPT 4.1) was used to assist with phrasing in Section 2 and Section 4. The authors reviewed the accuracy and took responsibility for the final text, complying with the SAGE Guidelines [10].

### 2.4. Risk of Bias

The risk of bias in the included studies was assessed using the Newcastle–Ottawa Scale for observational studies. Each study of the 43 included in the results was evaluated based on the three domains: selection of the study group (0–4 stars), the comparability of the groups (0–2 stars) and the outcomes (0–3 stars). Afterwards, studies were categorized as high quality (7–9 stars), moderate quality (4–6 stars), or low quality (0–3 stars).

The detailed quality assessment for each of the 43 studies is presented in Table 1.

It is also important to note that this review addresses a clinical domain in which the number of available cases is relatively small. Hereditary pancreatic cancer and genetically predisposed high-risk individuals represent a small subset of all pancreatic cancer cases, which inherently restricts the size and power of most individual studies, limiting the generalizability of the findings.

### 2.5. Effect Measures

For each included study, the main effects extracted were the relative risk (RR), odds ratio (OR), standardized incidence ratio (SIR), or hazard ratio (HR) within the 95% confidence intervals, if available. The cumulative lifetime risk was also retrieved from some studies. These measures were used to stratify the risk of pancreatic cancer in high-risk individuals.

To provide a clinical perspective to our findings, stratification based on the risk of developing pancreatic cancer for each syndrome was devised. We defined three risk categories: low risk (<5), moderate risk (5–10) and high risk (>10). This stratification should provide a better identification of groups that would benefit from a surveillance program.

### 2.6. Synthesis Method

Given the heterogeneity of the included studies, the population, and the measured outcomes, a meta-analysis was not feasible. Thus, a systematic review was conducted.

Results were reported using a structured table to highlight the key findings.

### 2.7. Certainty of Evidence

Based on the Oxford Centre for Evidence-Based Medicine 2009 framework, this systematic review provides level 2a evidence (considering the cohort studies included). However, there are many small, retrospective, and some case–control studies; therefore, the overall strength drops to level 3a [53].

## 3. Results

**Figure 1:**
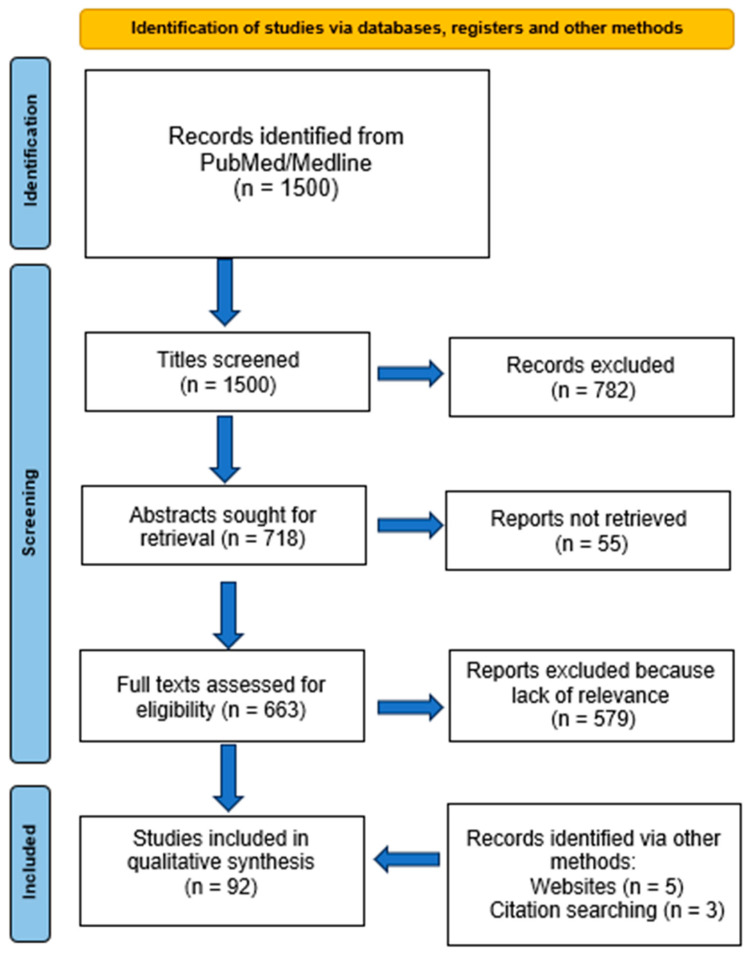
PRISMA flow diagram.

1500 articles were screened. Of these, 782 were excluded based on the title (mostly articles about neuroendocrine tumors), and 55 were not retrieved because of a lack of abstracts and full texts. Of the 663 remaining articles, 478 were excluded based on the lack of relevant data (PRISMA flow diagram reported in Figure 1).

Three articles were retrieved from different websites, and three articles were retrieved from the citations of the articles resulting from the search strategy. Finally, 92 articles were included.

The methodological quality of the 43 observational studies included in the results was evaluated using the Newcastle–Ottawa Scale. Overall, the studies demonstrated good quality, with a mean of 7.42 out of 9 stars.

Of the 42 studies, 33 were classified as high quality, showing a low risk of bias, while 2 were identified with a higher risk of bias, primarily because of a small sample size and unsatisfactory follow-up. The remaining eight studies were included in the moderate-risk category, mostly for an insufficient representativeness of the cases and adjustments for confounding factors.

### 3.1. Lynch Syndrome

Former hereditary nonpolyposis colorectal cancer consists of DNA mismatch repair genes mutations, mostly MLH1 and MSH2, but also MSH6, PMS2, and EPCAM. It is characterized by early onset colorectal cancer but is also associated with endometrial, ovarian, gastric, small intestine, urinary tract, brain, pancreatic, and cutaneous sebaceous glands. These genes encode proteins that bind to mismatched double-stranded DNA and microsatellites to prepare them for repair [54,55,56].

A total of 147 families with MMR gene mutations, comprising 6342 individuals, were included in a study to determine the risk of developing pancreatic cancer. The result was an 8.6-fold increase (95% CI, 4.7–15.7) compared with the general population. The risk was higher before age 50 (HR 30.5 for ages 20–49 years, 95% CI: 14.2, 65.7) and then decreased with increasing age (HR 5.1 for ages 50–70 years, 95% CI: 2.2, 11.8). Patients with MLH1 mutation had a lower risk than the ones with MSH2 (HR = 7.5, 95% CI: 2.4, 23.0 compared to HR = 10.9, 95% CI: 5.5, 21.9). MSH6 carriers included were too few, so the risk was not calculated [11].

Other studies have revealed a 4.5–4.7 risk of developing pancreatic cancer in patients with Lynch syndrome [12,13].

A 5-year follow-up of 446 unaffected carriers of MMR gene mutations found a risk of Standardized Infection Ratio of 10.68 (95% CI, 2.68 to 47.70; *p* = 0.001) [14].

Dowty et al. revealed that the risk of pancreatic cancer in Lynch Syndrome patients was different in males and females and was based on the mutated gene. MLH1 mutation carried a risk of 2.5 (CI 95%, 0.07–85.2) in males and 5.9 (CI 95%, 1.0–34.0) in females, while MSH2 carried a risk of 18.1 (CI 95%, 8.4–39.0, *p* < 0.001) and 4.7 (CI 95%, 1.1–19.6), respectively. The overall risk was calculated as 4.0 when compared to sporadic pancreatic cancer [15,57].

Mølle et al. researched the prevalence of different cancer in the four major mutations of Lynch syndrome and found out that the lifetime risk for pancreatic cancer was 0 for PMS2, 0.5% for MSH2 and 1.4% for MSH6 regardless of age. However, MLH1 patients had a similar risk as the general population up to 60 years of age. Afterwards, starting with the seventh decade, they had a 3.9% chance at 70 years and 6.2% chance at 75 years of being diagnosed with pancreatic cancer [16].

In a retrospective study of the Finland Lynch Syndrome database, the most frequent mutation was of MLH1, followed by MSH2, while in the MSH6 and PMS2 groups, no pancreatic cancers were detected [17]. A study of the Prospective Lynch Syndrome Database confirmed these findings [18,58].

### 3.2. Hereditary Breast-Ovarian Cancer

Hereditary breast-ovarian cancer (HBOC) is an autosomal dominant inherited disease characterized by early onset breast and/or ovarian cancers caused by mutations in the BRCA 1 and BRCA 2 genes. It typically includes patients with early onset (under 50 years) and at least two family members with breast and/or ovarian cancer [19,20].

The presence of BRCA1/2 mutations appears to be 18.9% in individuals at high risk of developing pancreatic cancer and 21.9% in patients with a history of pancreatic cancer [59].

A cohort study of family members of patients with breast cancer found that first-degree relatives have a higher risk of pancreatic cancer (RR = 1.66 for males and RR = 1.39 for females), with the risk decreasing in lower grade relatives. The weighted RR was 1.3 for all family members (both statistically significant) [60].

A study of 11,847 patients revealed that BRCA 1 mutations carry a RR of 2.26 (95% [CI] = 1.26–4.06, *p* = 0.004) of developing pancreatic cancer [21], while Lener et al. genotyped 400 familial pancreatic cancer individuals and found out that the risk of developing pancreatic cancer in BRCA 1 mutation individuals was 6.72 (CI 95% 1.94–23.30, *p* = 0.006) [22].

A review found that BRCA 1 mutation individuals carry a 2.3 to 3.6-fold increased risk of PC and those of BRCA2 may carry as much as a 3 to 10-fold increased risk of PC [61].

Patients with BRCA 2 mutation, by the age of 75, have a cumulative risk of about 7% (95% CI, 1.9–19%) of developing pancreatic cancer, while the general population has a risk of 0.85% [62]. A study of 49 families with breast cancer showed that BRCA2 mutation was associated with an increased risk of pancreatic cancer (RR 7.2; *p* = 0.03) [23]. It seems that patients with BRCA 2 mutations carry a 10-fold risk of developing pancreatic cancer throughout their lifetimes [24,63], while another study indicates a RR of 3.51 (95% CI: 1.9–6.6) [6].

A study of 1072 individuals with BRCA mutations revealed that BRCA 1 mutation was not associated with an increased risk of pancreatic cancer, while individuals with BRCA 2 mutation had a higher incidence of pancreatic cancer than expected (SIR 21.745, 95% CI 13.086–33.96, *p* < 0.001). Furthermore, the incidence was higher in males (SIR 82.559, 95% CI 39.524–151.84, *p* < 0.001) than in females (SIR 13.809, 95% CI 6.301–26.216, *p* < 0.001) [25].

### 3.3. Peutz–Jeghers Syndrome

Peutz–Jeghers syndrome is an autosomal dominant disease with variable penetrance, best known for hamartomatous polyps in the gastrointestinal tract, especially in the colon, and mucocutaneous pigmented lesions. It is caused by a mutation in the STK11 gene and is associated with an increased risk of digestive and extra-digestive cancers, including pancreatic cancer [61,64]. One study showed that the risk was as high as 132 (CI 95% = 44–261, *p* < 0.001). The risk of developing pancreatic cancer from 15 to 64 years is 36% (third place after breast cancer [54%] and colon cancer [39%]), with a cumulative risk of 93% [26].

An Italian multicenter study including 119 PJS patients found an RR of 139.7 (CI 61.1–276.4) for developing pancreatic cancer compared to the general population. This study has also revealed that the risk was much higher in female individuals (245.4, CI 95% = 78.0–591.9) than in males (88.6, CI 95% 22.6–241.6) [27,28].

The risk of pancreatic cancer in individuals carrying this mutation is 3%, 5%, 7%, and 11% by the age of 40, 50, 60, and 70 years, respectively [29].

### 3.4. Li-Fraumeni Syndrome

Li-Fraumeni Syndrome is produced by a germline pathogenic variant in the TP53 gene and it is associated with an increased risk of pancreatic cancer [65].

Ruijs et al. investigated 180 Dutch families with TP53 mutations and revealed that the relative risk of developing pancreatic cancer was 7.3 (CI 95% = 2–19, *p* = 0.006) [14,30], while others place the risk at under 5% [27].

### 3.5. Familial Adenomatous Polyposis

Familial adenomatous polyposis is a highly penetrant autosomal dominant disease characterized by the presence of hundreds to thousands of adenomatous polyps throughout the gastrointestinal tract, with more polyps appearing as the patient ages. It is caused by mutations in the APC gene [66].

A study of 197 familial adenomatous polyposis pedigrees found an RR of 4.46 (95% CI 1.2–11.4) for pancreatic adenocarcinoma [31], while another study reported the same result, with an RR of 5 [24].

Gardner syndrome, a subtype of autosomal dominant familial adenomatous polyposis, carries a significant risk of developing periampullary malignancies. This risk indicates the need for periodic screening through upper gastrointestinal tract endoscopy [67,68].

The duplication of the small arm of chromosome 7, containing the gene SKAP2, is associated with an increased risk of pancreatic ductal adenocarcinoma; however, the risk is unknown [69].

### 3.6. Ataxia Telangiectasia Syndrome

The mutation of the DNA repair gene ATM, known as ataxia telangiectasia syndrome, was shown in a US retrospective cohort covering 6 years that the risk for pancreatic cancer was the highest among the researched cancer types (OR 4.21, 95% CI 3.24–5.47) [32], while an Italian study revealed a 3-fold risk [12]. Other studies have only shown an increased risk among close relatives [20,31].

Perez et al. found a relative risk of 2.70 (CI 95%, 0.87–6.31) [33], while a large case–control study of pancreatic cancer patients found that the risk of developing pancreatic cancer in ATM mutation individuals was 5.71 (95% CI = 4.38–7.33) [34].

Another large retrospective study found an association between this mutation and pancreatic cancer, with an OR of 4.44 (95% CI, 2.66–7.40) [35].

A relative risk of 6.5 (95% CI, 4.5–9.5) was found in a US/Canadian cohort study [36,37].

### 3.7. Von Hippel–Lindau Disease

von Hippel–Lindau Disease, produced by the mutation of the VHL gene, is associated with an increased risk of pancreatic neuroendocrine and exocrine tumors [38,39,40]. However, in most patients, the disease is associated with pancreatic cysts, with a prevalence of 50–91% [70], which require frequent surveillance [54,55]. Considering the frequent lesions that appear in these patients, a study revealed that EUS found similar lesions in family members confirmed with the same mutation [71].

A case report series of 23 patients with VHL screened with MRI revealed that all patients had pancreatic lesions, ranging from cystic lesions and serous cystadenomas to neuroendocrine tumors, but no adenocarcinoma was found [72].

### 3.8. Familial Pancreatic Cancer

Familial pancreatic cancer is defined as a cluster of pancreatic cancer cases in first-degree relatives, excluding more general cancer syndromes. It specifically refers to the predisposition to pancreatic cancer and usually involves at least two kindreds [73,74]. It is believed that these patients carry an unknown autosomal dominant mutation with 80% penetrance [75].

In families with an aggregation of pancreatic cancer, with at least three first-degree relatives diagnosed, the risk of another member developing pancreatic cancer increased up to 57-fold over the general population risk [76]. Another study found that the risk of developing pancreatic cancer in close relatives of a pancreatic cancer patient was OR = 5.25 (95% CI = 2.08–13.21) [77]. Moreover, an analysis of 362 relatives found that the risk of pancreatic cancer was higher if there are two second-degree relatives than a single member (3.7% versus 0.6%, *p* < 0.0001) [63]. Another study has shown that the risk of developing pancreatic cancer in cluster families is 18-fold (95% CI = 4.74–44.5) if a first-degree relative was diagnosed and 57-fold (95% CI = 12.4–175) if three or more relatives are affected [41].

An Italian case–control study of 362 pancreatic cancer patients showed that a family history of pancreatic cancer carries a 3-fold individual risk (95% CI, 1.4 to 6.6) [78]. Another case–control study of 247 probands found an RR of 2.49 (95% [CI] = 1.32–4.69) for developing pancreatic cancer with a positive family history [79], while another case–control returned a value of 2.7 (1.7–4.3) [80,81].

First-degree relatives of patients with pancreatic cancer have a risk of 1.88 (95% CI = 1.27–2.68) of developing the same malady [42], while children of parents with pancreatic cancer have a standardized incidence ratio of 1.73 (95% CI 1.13–2.54) for developing pancreatic adenocarcinoma [43,82].

When considering distant relatives, the risk of developing pancreatic cancer is 1.28 with one or more affected second-degree relatives (with a lifetime risk of 1.70%), 1.09 with one third-degree relative affected (1.40% lifetime risk), and 1.06 with one fourth-degree relative (1.4% lifetime risk). The risk in the general population is 1, with a lifetime risk of 1.30% [61].

The risk of pancreatic cancer in patients with a familial history was elevated in individuals with three (32.0; 95% CI, 10.2–74.7), two (6.4; CI, 1.8–16.4), or one (4.6; CI, 0.5–16.4) first-degree relative(s) with pancreatic cancer [44].

A Johns Hopkins Hospital study over a 12-year period, including 9040 individuals who had at least one first-degree relative with pancreatic cancer, found that the risk of developing the cancer varied by the number of first-degree relatives with pancreatic cancer: 17.02 (95% CI = 7.34 to 33.5, *p* < 0.001) for individuals with three first-degree relatives, 3.9 (95% CI = 1.59 to 8.2, *p* = 0.005) with two first-degree relatives, and 6.86 (95% CI = 3.75 to 11.04, *p* < 0.001) with one, with a mean of 6.79 (95% CI = 4.54 to 9.75, *p* < 0.001). The risk was 2.41 (95% CI = 1.04 to 4.74, *p* = 0.04) in cases of kindred with sporadic pancreatic cancer [45].

A pooled analysis of six studies (five cohorts and one case–control) found a risk of 1.76 (95% CI = 1.19–2.61) if one first-degree relative was diagnosed. The risk was higher in case of two, but was not statistically relevant (4.26, 95% CI = 0.48–37.79) [83].

One of the larger studies, with 484 pancreatic cancer cases and 2099 controls, in the USA found out that pancreatic cancer patients reported a first-degree relative with pancreatic cancer more often than controls with an OR of 3.2 (95% CI = 1.8–5.6), and the risk was higher with an OR of 3.6 (95% CI = 1.5–8.7), among those with an affected sibling compared to those with an affected parent with an OR = 2.6 (95% CI = 1.2–5.4) [19,46].

### 3.9. Familial Atypical Multiple Mole Melanoma Syndrome

Familial Atypical Multiple Mole Melanoma syndrome (FAMMM) is an autosomal dominant disease caused by mutations in CDKN2A and is characterized by multiple melanocytic nevi, usually more than 50, and a family history of melanoma [84]. A study of 19 families with known melanoma in at least two first-degree relatives has shown that this mutation carries an increased risk of developing pancreatic cancer (RR = 21.8; 95% CI = 8.7–44.8) [47]. Another study of 9 families with 200 members found a similar result (OR = 13.4 with *p* < 0.001) [31]. The estimated cumulative risk of developing pancreatic cancer by the age of 75 years was 17% [48]. One study has revealed that the risk of developing pancreatic cancer in this syndrome is from 13 fold to 22 [24], while another study shows a risk as high as 47.8 (95% CI = 28.4–74.7) [49,85].

### 3.10. Hereditary Pancreatitis

Hereditary pancreatitis is an autosomal dominant disease characterized by recurrent episodes of acute pancreatitis, with a usual debut in the first two decades of life. It is caused by mutations in genes that code for pancreatic enzymes, such as PRSS1, SPINK1, CFTR, and CTRC [86], and carries a 53-fold increased risk of developing pancreatic cancer [50].

A Japanese consortium review found a 50–70 risk [8]. Lowenfels et al. found a similar result, a 50–60 fold risk compared to the general population [51].

Moreover, another study found a SIR of 87 (95% CI 42–113), with 69 (25–150) in men and 142 (38–225) in women [52].

A summary of the results, with the risk of developing pancreatic cancer in each genetic syndrome, can be found in Table 2.

## 4. Discussion

This systematic review highlights the important role of genetic components in the development of pancreatic cancer, especially in familial clusters or specific gene mutations. Our findings support the fact that a subset of pancreatic cancers arises from inherited predisposition, often involving high-penetrance mutations such as BRCA1 and BRCA2 (hereditary breast and ovarian cancer), CDKN2A (Familial Atypical Multiple Mole Melanoma syndrome), STK11 (PeutzJeghers syndrome), and PRSS1 and SPINK1 (hereditary pancreatitis).

The methodological quality of the studies included in the present review was evaluated using the Newcastle–Ottawa Scale. Overall, the evidence demonstrated a high level of scientific integrity, with a mean score of 7.42 out of 9 stars.

Most studies [33] were classified as high quality (7–9 stars). They had a good cohort selection, clear definitions of genetic predisposition, and reliable methods for confirming pancreatic cancer (e.g., histopathological).

On the other hand, only two studies were found to be of low quality (0–3 stars), having a high risk of bias.

Our review also revealed that familial pancreatic cancer (usually defined as at least two first-degree relatives without other known germline mutations) is a clinically significant entity but is poorly understood, especially considering the lack of data regarding specific gene mutations.

Pancreatic cancer is linked to a wide variety of genetic syndromes, suggesting multiple neoplastic pathways. This alone highlights the need for genetic counseling and surveillance strategies for each patient and their families. A screening program may be beneficial for individuals at high risk of developing pancreatic cancer. In addition, multigene panel testing should be considered, as prices decrease over time.

Germline testing is typically reserved for individuals meeting specific criteria, based on family history or age of onset. However, our findings align with recent evidence suggesting that family history alone is enough for genetic screening. Further research should transition from a gene-specific focus to a genome view. The development of polygenic risk scores, which aggregate the effects of multiple low-penetrance variants, hold the potential to further refine risk stratification. Additionally, the integration of liquid biopsy techniques, focusing on circulating tumor DNA, may provide a less invasive tool for monitoring such patients. All this data can be combined with clinical data by emerging artificial intelligence algorithms to further enhance the precision of surveillance programs.

We stratified every syndrome based on the risk of pancreatic cancer (Table 3). We decided to include two thresholds, with three categories. A relative risk of 5 or lower was defined as low risk. We included familial adenomatous polyposis and von Hippel–Lindau Syndrome as low risk.

Moderate risk [5,6,7,8,9,10] includes hereditary breast and ovarian cancer syndrome (specifically the BRCA 1 mutation), Lynch Syndrome, Li Fraumeni Syndrome, Ataxia Teleangiectasia and patients from familial pancreatic cancer clusters with 1–2 affected kindreds.

Finally, we classified individuals with Peutz–Jeghers Syndrome, Hereditary Pancreatitis, Familial Atypical Multiple Melanoma Syndrome, familial pancreatic cancer (at least three affected kindreds) and hereditary breast and ovarian cancer syndrome with BRCA 2 mutation as high risk.

Note that we decided to establish two thresholds and include three risk-based categories. This division should make it easier to choose a target population if a future-screening program would be established. Given the high cost of a surveillance program (with the possibility of invasive procedure like endoscopic ultrasound or retrograde endoscopic cholangiopancreatography), a screening population should be carefully defined. This way, at least high-risk individuals should be included in a screening program. Moderate-risk patients might also benefit.

The 2013 CAPS consortium guidelines establishes a threshold for high-risk individuals to be screened (>5% lifetime risk or five-fold increased relative risk) [92].

The latest ESMO guidelines recommend that high-risk individuals should undergo yearly screening, with endoscopic ultrasound and/or pancreatic magnetic resonance imaging. The surveillance should begin at age 50 (or 10 years earlier than the age of the youngest affected relative) [93].

A special mention should be made concerning the difference in values in some studies. While most values are within the same range, there are some exceptions. In BRCA 2 mutations, one study has shown a much higher risk (21.7) compared to the others [25]. This is primary caused by heterogeneity of the study population and study design. Furthermore, values that are well outside the range are typically derived from smaller cohorts.

Overall, this review reiterates that identifying individuals at high risk of developing pancreatic cancer has the potential for an earlier diagnosis with targeted surveillance. This may also guide ulterior treatment strategies, especially as oncology treatments evolve. The data synthesized in this review have several important limitations. First, all studies included in this review were observational, with most being retrospective cohorts. This is associated with a moderate-to-high risk of selection bias because of the lack of randomization. Secondly the low prevalence of pancreatic cancer and, even more scarcely, the number of cases of pancreatic cancer with an inherited gene mutation, limit the sample size. Third, while the search was limited to PubMed, given its extensive medical literature, we acknowledge that the omission of other databases like Embase or Web of Science might have resulted in the exclusion of some relevant articles.

## 5. Conclusions

It might be beneficial to identify individuals at increased risk for developing pancreatic cancer who may benefit from genetic counseling, genetic testing, and potential inclusion in a screening program. All these are for the purpose of early diagnosis and improved outcomes. Future research should focus on a large prospective cohort, screening programs, and emerging technologies such as AI-assisted imaging and work-up. The highest risk of developing pancreatic cancer due to inherited genetic mutations and familial aggregation was found in Peutz–Jeghers syndrome, hereditary pancreatitis, Familial Atypical Multiple Mole Melanoma syndrome, hereditary breast and ovarian cancer with BRCA2 mutation, and familial pancreatic cancer with at least three affected kindreds. Moderate risk was associated with BRCA1, MLH1, MSH2, MSH6, p53, and ATM mutations, and familial pancreatic cancer with 1–2 affected kindred. Low risk was found in familial adenomatous polyposis and von Hippel–Lindau Syndrome.

## Figures and Tables

**Table 1:** Quality assessment of the included studies according to the Newcastle–Ottawa Scale.

Study (Author, Year)	Selection (0–4 Stars)	Compatibility (0–2 Stars)	Outcome (0–3 Stars)	Total Score	Quality/Risk of Bias
Wada et al. (2013) [8]	***	*	**	6/9	Moderate Quality
Kastrinos et al. (2009) [11]	****	*	*	6/9	Moderate Quality
Del Chiaro et al. (2010) [12]	***	*	***	7/9	High Quality
Aarnio et al. (1999) [13]	****	*	***	8/9	High Quality
Win et al. (2012) [14]	****	**	***	9/9	High Quality
Dowty et al. (2013) [15]	****	**	***	9/9	High Quality
Moller at al. (2018) [16]	****	**	***	9/9	High Quality
Zalevskaja et al. (2023) [17]	***	*	***	7/9	High Quality
Dominquez-Valentin et al. (2023) [18]	****	**	***	9/9	High Quality
Matsubayashi et al. (2011) [19]	****	**	***	9/9	High Quality
LaDuca et al. (2020) [20]	****	**	***	9/9	High Quality
Thompson et al. (2002) [21]	****	*	***	8/9	High Quality
Lener at al. (2017) [22]	***	*	***	7/9	High Quality
Phelan et al. (1996) [23]	**	*	**	5/9	Moderate Quality
Lynch et al. (2005) [24]	**		**	4/9	Moderate Quality
Mersch et al. (2015) [25]	****	*	***	8/9	High Quality
Giardiello et al. (2000) [26]	****	**	***	9/9	High Quality
Schneider et al. (2011) [27]	****	*	***	8/9	High Quality
Resta et al. (2013) [28]	***	**	**	6/9	Moderate Quality
Hearle et al. (2006) [29]	****	**	**	8/9	High Quality
Ruijs et al. (2010) [30]	***	*	***	7/9	High Quality
Flanders et al. (1996) [31]	**		*	3/9	Low Quality
Hall et al. (2021) [32]	****	**	***	9/9	High Quality
Geoffroy-Perez et al. (2001) [33]	***	*	**	6/9	Moderate Quality
Hu et al.l. (2018) [34]	****	**	***	9/9	High Quality
Zeng et al. (2022) [35]	****	**	***	9/9	High Quality
Wang et al. (2022) [36]	***	*	***	7/9	High Quality
Hsu et al. (2021) [37]	***	*	***	7/9	High Quality
Pasternak-Pietrzak. (2024) [38]	**	*	**	5/9	Moderate Quality
Fill et al. (1979) [39]	*	*	*	3/9	Low Quality
Glavac et al. (1996) [40]	***	*	***	7/9	High Quality
Tersmette et al. (2001) [41]	****	**	***	9/9	High Quality
McWilliams at al. (2005) [42]	****	**	**	8/9	High Quality
Hemminki et al. (2003) [43]	****	**	***	9/9	High Quality
Klein et al. (2004) [44]	****	**	***	9/9	High Quality
Brune et al. (2010) [45]	****	**	***	9/9	High Quality
Silverman et al. (1999) [46]	****	**	**	8/9	High Quality
Goldstein et al. (1995) [47]	***	*	**	6/9	Moderate Quality
Vasen et al. (2000) [48]	****	**	***	9/9	High Quality
de Snoo et al. (2008) [49]	****	**	***	9/9	High Quality
Lowenfels et al. (1997) [50]	****	*	***	8/9	High Quality
Lowenfels et al. (2000) [51]	****	**	***	9/9	High Quality
Rebours et al. (2008) [52]	****	**	***	9/9	High Quality

A maximum of 9 stars (*) can be achieved. Selection (max. 4 stars), compatibility (max. 2 stars) and outcome (max. 3 stars). Studies scoring 7–9 stars are considered high quality, 4–6 stars moderate quality and 0–3 stars low quality.

**Table 2:** Risk of pancreatic cancer in different genetic syndromes.

Syndrome	Gene Mutations (Locus)	Relative Risk	Lifetime Risk (%)	References
Peutz Jeghers syndrome	STK11 (19p13.3)	132–140	11–36 by the age of 70	[26,27,28,29]
Hereditary pancreatitis	PRSS1 (7q35) SPINK1 (5q31)P	50–87	25–40 by the age of 70	[8,50,51,52,86]
Familial Atypical Multiple Mole Melanoma syndrome (FAMMM)	CDKN2A (9p21)	13.4–47.8	17	[24,31,47,48,49,85,87]
Hereditary breast and ovarian cancer	BRCA 2 (13q12) BRCA 1 (17q21)	3–21.7 2.3–6.7	1–3% 2–7%	[7,21,22,23,24,25,61,62,63,88]
Lynch syndrome	MLH1 (3p21), MSH2 (2p21–22), MSH6	4–10.6	3.7 by age 70	[11,12,13,14,15,16,17,18,57,58,89]
Li Fraumeni syndrome	p53	7.3	<5	[14,27,30,90]
Familial adenomatous polyposis	APC (5q21)	4.5	1.7 by the age of 80	[24,31,91]
Ataxia Telangiectasia	ATM (11q22–23)	2.7–6.5	6.3 by the age of 70	[12,20,31,32,33,34,35,36,37]
Familial pancreatic cancer 1 FDR 2 FDR 3 FDR	Mostly unknown	1.7–18 3.9–6.4 17–57	6 8–12 40	[19,41,42,43,44,45,46,76,77,82,87]
von Hippel–Lindau	VHL (3p25)	<5	Unknown	[38,39,40]

**Table 3:** Proposed risk stratification based on cumulative risk for pancreatic cancer.

Risk Category	Lifetime Risk	Hereditary Syndrome/Mutation
Low Risk	<5	Familial Adenomatous Polyposis, von Hippel–Lindau Syndrome
Moderate Risk	5–10	Hereditary Breast and Ovarian Cancer Syndrome (BRCA 1 mutation), Lynch Syndrome, Li Fraumeni Syndrome, Ataxia Teleangiectasia, Familial Pancreatic Cancer (1–2 affected kindreds)
High Risk	>10	Peutz–Jeghers, Hereditary Pancreatitis, Familial Atypical Multiple Melanoma Syndrome, Familial Pancreatic Cancer (at least three affected kindreds), Hereditary Breast and Ovarian Cancer Syndrome (BRCA 2 mutation)

## Data Availability

All data generated or analyzed during this study are included in this article. Further enquiries can be directed to the corresponding authors.

## Supplementary Materials

The following supporting information can be downloaded at: https://www.mdpi.com/article/10.3390/cancers18060976/s1, Table S1: PRISMA Checklist. Reference [9] is cited in the supplementary materials.

## References

[1] Cancer Today. https://gco.iarc.who.int/today/.

[2] SURVMARK-2-Viz2. https://gco.iarc.fr/survival/survmark/visualizations/viz2/?cancer_site=%22Pancreas%22&country=%22United+Kingdom%22&agegroup=%22All%22&gender=%22All%22&interval=%221%22&survival_year=%221%22&measures=%5B%22Incidence+%28ASR%29%22%2C%22Mortality+%28ASR%29%22%2C%22Net+Survival%22%5D.

[3] Lynch H.T., Deters C.A., Lynch J.F., Brand R.A. (2002). Challenging Pancreatic Cancer-Prone Pedigrees: A Nosologic Dilemma. Am. J. Gastroenterol..

[4] Klein A.P. (2012). Genetic Susceptibility to Pancreatic Cancer. Mol. Carcinog..

[5] Goggins M., Overbeek K.A., Brand R., Syngal S., Del Chiaro M., Bartsch D.K., Bassi C., Carrato A., Farrell J., Fishman E.K. (2020). Management of Patients with Increased Risk for Familial Pancreatic Cancer: Updated Recommendations from the International Cancer of the Pancreas Screening (CAPS) Consortium. Gut.

[6] Tempero M.A., Malafa M.P., Al-Hawary M., Behrman S.W., Benson A.B., Cardin D.B., Chiorean E.G., Chung V., Czito B., Del Chiaro M. (2021). Pancreatic Adenocarcinoma, Version 2.2021, NCCN Clinical Practice Guidelines in Oncology. J. Natl. Compr. Canc. Netw..

[7] Lynch H.T., Fusaro R.M., Lynch J.F. (2007). Hereditary Cancer Syndrome Diagnosis: Molecular Genetic Clues and Cancer Control. Future Oncol..

[8] Wada K., Takaori K., Traverso L.W., Hruban R.H., Furukawa T., Brentnall T.A., Hatori T., Sano K., Takada T., Majima Y. (2013). Clinical Importance of Familial Pancreatic Cancer Registry in Japan: A Report from Kick-off Meeting at International Symposium on Pancreas Cancer 2012. J. Hepatobiliary Pancreat. Sci..

[9] Page M.J., McKenzie J.E., Bossuyt P.M., Boutron I., Hoffmann T.C., Mulrow C.D., Shamseer L., Tetzlaff J.M., Akl E.A., Brennan S.E. (2021). The PRISMA 2020 Statement: An Updated Guideline for Reporting Systematic Reviews. BMJ.

[10] Assistive and Generative AI Guidelines for Authors. https://www.sagepub.com/about/policies/ai-author-guidelines?utm_source=chatgpt.com.

[11] Kastrinos F., Mukherjee B., Tayob N., Wang F., Sparr J., Raymond V.M., Bandipalliam P., Stoffel E.M., Gruber S.B., Syngal S. (2009). Risk of Pancreatic Cancer in Families with Lynch Syndrome. JAMA.

[12] Del Chiaro M., Zerbi A., Capurso G., Zamboni G., Maisonneuve P., Presciuttini S., Arcidiacono P.G., Calculli L., Falconi M., Italian Registry for Familial Pancreatic Cancer Familial Pancreatic Cancer in Italy (2010). Risk Assessment, Screening Programs and Clinical Approach: A Position Paper from the Italian Registry. Dig. Liver Dis..

[13] Aarnio M., Sankila R., Pukkala E., Salovaara R., Aaltonen L.A., de la Chapelle A., Peltomäki P., Mecklin J.P., Järvinen H.J. (1999). Cancer Risk in Mutation Carriers of DNA-Mismatch-Repair Genes. Int. J. Cancer.

[14] Win A.K., Young J.P., Lindor N.M., Tucker K.M., Ahnen D.J., Young G.P., Buchanan D.D., Clendenning M., Giles G.G., Winship I. (2012). Colorectal and Other Cancer Risks for Carriers and Noncarriers from Families with a DNA Mismatch Repair Gene Mutation: A Prospective Cohort Study. J. Clin. Oncol..

[15] Dowty J.G., Win A.K., Buchanan D.D., Lindor N.M., Macrae F.A., Clendenning M., Antill Y.C., Thibodeau S.N., Casey G., Gallinger S. (2013). Cancer Risks for MLH1 and MSH2 Mutation Carriers. Hum. Mutat..

[16] Møller P., Seppälä T.T., Bernstein I., Holinski-Feder E., Sala P., Gareth Evans D., Lindblom A., Macrae F., Blanco I., Sijmons R.H. (2018). Cancer Risk and Survival in Path_MMR Carriers by Gene and Gender up to 75 Years of Age: A Report from the Prospective Lynch Syndrome Database. Gut.

[17] Zalevskaja K., Mecklin J.-P., Seppälä T.T. (2023). Clinical Characteristics of Pancreatic and Biliary Tract Cancers in Lynch Syndrome: A Retrospective Analysis from the Finnish National Lynch Syndrome Research Registry. Front. Oncol..

[18] Dominguez-Valentin M., Haupt S., Seppälä T.T., Sampson J.R., Sunde L., Bernstein I., Jenkins M.A., Engel C., Aretz S., Nielsen M. (2023). Mortality by Age, Gene and Gender in Carriers of Pathogenic Mismatch Repair Gene Variants Receiving Surveillance for Early Cancer Diagnosis and Treatment: A Report from the Prospective Lynch Syndrome Database. EClinicalMedicine.

[19] Matsubayashi H. (2011). Familial Pancreatic Cancer and Hereditary Syndromes: Screening Strategy for High-Risk Individuals. J. Gastroenterol..

[20] LaDuca H., Polley E.C., Yussuf A., Hoang L., Gutierrez S., Hart S.N., Yadav S., Hu C., Na J., Goldgar D.E. (2020). A Clinical Guide to Hereditary Cancer Panel Testing: Evaluation of Gene-Specific Cancer Associations and Sensitivity of Genetic Testing Criteria in a Cohort of 165,000 High-Risk Patients. Genet. Med..

[21] Thompson D., Easton D.F. (2002). Breast Cancer Linkage Consortium Cancer Incidence in BRCA1 Mutation Carriers. J. Natl. Cancer Inst..

[22] Lener M.R., Kashyap A., Kluźniak W., Cybulski C., Soluch A., Pietrzak S., Huzarski T., Gronwald J., Lubiński J. (2017). The Prevalence of Founder Mutations among Individuals from Families with Familial Pancreatic Cancer Syndrome. Cancer Res. Treat..

[23] Phelan C.M., Lancaster J.M., Tonin P., Gumbs C., Cochran C., Carter R., Ghadirian P., Perret C., Moslehi R., Dion F. (1996). Mutation Analysis of the BRCA2 Gene in 49 Site-Specific Breast Cancer Families. Nat. Genet..

[24] Lynch H.T., Deters C.A., Snyder C.L., Lynch J.F., Villeneuve P., Silberstein J., Martin H., Narod S.A., Brand R.E. (2005). BRCA1 and Pancreatic Cancer: Pedigree Findings and Their Causal Relationships. Cancer Genet. Cytogenet..

[25] Mersch J., Jackson M.A., Park M., Nebgen D., Peterson S.K., Singletary C., Arun B.K., Litton J.K. (2015). Cancers Associated WithBRCA1andBRCA2mutations Other than Breast and Ovarian: BRCAand Other Cancers. Cancer.

[26] Giardiello F.M., Brensinger J.D., Tersmette A.C., Goodman S.N., Petersen G.M., Booker S.V., Cruz-Correa M., Offerhaus J.A. (2000). Very High Risk of Cancer in Familial Peutz-Jeghers Syndrome. Gastroenterology.

[27] Schneider R., Slater E.P., Sina M., Habbe N., Fendrich V., Matthäi E., Langer P., Bartsch D.K. (2011). German National Case Collection for Familial Pancreatic Cancer (FaPaCa): Ten Years Experience. Fam. Cancer.

[28] Resta N., Pierannunzio D., Lenato G.M., Stella A., Capocaccia R., Bagnulo R., Lastella P., Susca F.C., Bozzao C., Loconte D.C. (2013). Cancer Risk Associated with STK11/LKB1 Germline Mutations in Peutz-Jeghers Syndrome Patients: Results of an Italian Multicenter Study. Dig. Liver Dis..

[29] Hearle N., Schumacher V., Menko F.H., Olschwang S., Boardman L.A., Gille J.J.P., Keller J.J., Westerman A.M., Scott R.J., Lim W. (2006). Frequency and Spectrum of Cancers in the Peutz-Jeghers Syndrome. Clin. Cancer Res..

[30] Ruijs M.W.G., Verhoef S., Rookus M.A., Pruntel R., van der Hout A.H., Hogervorst F.B.L., Kluijt I., Sijmons R.H., Aalfs C.M., Wagner A. (2010). TP53 Germline Mutation Testing in 180 Families Suspected of Li-Fraumeni Syndrome: Mutation Detection Rate and Relative Frequency of Cancers in Different Familial Phenotypes. J. Med. Genet..

[31] Flanders T.Y., Foulkes W.D. (1996). Pancreatic Adenocarcinoma: Epidemiology and Genetics. J. Med. Genet..

[32] Hall M.J., Bernhisel R., Hughes E., Larson K., Rosenthal E.T., Singh N.A., Lancaster J.M., Kurian A.W. (2021). Germline Pathogenic Variants in the Ataxia Telangiectasia Mutated (ATM) Gene Are Associated with High and Moderate Risks for Multiple Cancers. Cancer Prev. Res..

[33] Geoffroy-Perez B., Janin N., Ossian K., Laugé A., Croquette M.F., Griscelli C., Debré M., Bressac-de-Paillerets B., Aurias A., Stoppa-Lyonnet D. (2001). Cancer Risk in Heterozygotes for Ataxia-Telangiectasia. Int. J. Cancer.

[34] Hu C., Hart S.N., Polley E.C., Gnanaolivu R., Shimelis H., Lee K.Y., Lilyquist J., Na J., Moore R., Antwi S.O. (2018). Association between Inherited Germline Mutations in Cancer Predisposition Genes and Risk of Pancreatic Cancer. JAMA.

[35] Zeng C., Bastarache L.A., Tao R., Venner E., Hebbring S., Andujar J.D., Bland S.T., Crosslin D.R., Pratap S., Cooley A. (2022). Association of Pathogenic Variants in Hereditary Cancer Genes with Multiple Diseases. JAMA Oncol..

[36] Wang Y., Cuggia A., Chen Y.-I., Parent J., Stanek A., Denroche R.E., Zhang A., Grant R.C., Domecq C., Golesworthy B. (2022). Is Biannual Surveillance for Pancreatic Cancer Sufficient in Individuals with Genetic Syndromes or Familial Pancreatic Cancer?. J. Natl. Compr. Canc. Netw..

[37] Hsu F.-C., Roberts N.J., Childs E., Porter N., Rabe K.G., Borgida A., Ukaegbu C., Goggins M.G., Hruban R.H., Zogopoulos G. (2021). Risk of Pancreatic Cancer among Individuals with Pathogenic Variants in the ATM Gene. JAMA Oncol..

[38] Pasternak-Pietrzak K., Kozłowska A., Moszczyńska E. (2024). Hereditary Pheochromocytoma as a Main Manifestation of von Hippel Lindau Disease (VHL) in Childhood—A Long-Term Follow-up of 5 Patients with VHL from One Family. J. Clin. Res. Pediatr. Endocrinol..

[39] Fill W.L., Lamiell J.M., Polk N.O. (1979). The Radiographic Manifestations of von Hippel-Lindau Disease. Radiology.

[40] Glavac D., Neumann H.P., Wittke C., Jaenig H., Masek O., Streicher T., Pausch F., Engelhardt D., Plate K.H., Höfler H. (1996). Mutations in the VHL Tumor Suppressor Gene and Associated Lesions in Families with von Hippel-Lindau Disease from Central Europe. Hum. Genet..

[41] Tersmette A.C., Petersen G.M., Offerhaus G.J., Falatko F.C., Brune K.A., Goggins M., Rozenblum E., Wilentz R.E., Yeo C.J., Cameron J.L. (2001). Increased Risk of Incident Pancreatic Cancer among First-Degree Relatives of Patients with Familial Pancreatic Cancer. Clin. Cancer Res..

[42] McWilliams R.R., Rabe K.G., Olswold C., De Andrade M., Petersen G.M. (2005). Risk of Malignancy in First-Degree Relatives of Patients with Pancreatic Carcinoma. Cancer.

[43] Hemminki K., Li X. (2003). Familial and Second Primary Pancreatic Cancers: A Nationwide Epidemiologic Study from Sweden. Int. J. Cancer.

[44] Klein A.P., Brune K.A., Petersen G.M., Goggins M., Tersmette A.C., Offerhaus G.J.A., Griffin C., Cameron J.L., Yeo C.J., Kern S. (2004). Prospective Risk of Pancreatic Cancer in Familial Pancreatic Cancer Kindreds. Cancer Res..

[45] Brune K.A., Lau B., Palmisano E., Canto M., Goggins M.G., Hruban R.H., Klein A.P. (2010). Importance of Age of Onset in Pancreatic Cancer Kindreds. J. Natl. Cancer Inst..

[46] Silverman D.T., Schiffman M., Everhart J., Goldstein A., Lillemoe K.D., Swanson G.M., Schwartz A.G., Brown L.M., Greenberg R.S., Schoenberg J.B. (1999). Diabetes Mellitus, Other Medical Conditions and Familial History of Cancer as Risk Factors for Pancreatic Cancer. Br. J. Cancer.

[47] Goldstein A.M., Fraser M.C., Struewing J.P., Hussussian C.J., Ranade K., Zametkin D.P., Fontaine L.S., Organic S.M., Dracopoli N.C., Clark W.H. (1995). Increased Risk of Pancreatic Cancer in Melanoma-Prone Kindreds with P16INK4 Mutations. N. Engl. J. Med..

[48] Vasen H.F., Gruis N.A., Frants R.R., van Der Velden P.A., Hille E.T., Bergman W. (2000). Risk of Developing Pancreatic Cancer in Families with Familial Atypical Multiple Mole Melanoma Associated with a Specific 19 Deletion of P16 (P16-Leiden). Int. J. Cancer.

[49] de Snoo F.A., Bishop D.T., Bergman W., van Leeuwen I., van der Drift C., van Nieuwpoort F.A., Out-Luiting C.J., Vasen H.F., ter Huurne J.A.C., Frants R.R. (2008). Increased Risk of Cancer Other than Melanoma in CDKN2A Founder Mutation (P16-Leiden)-Positive Melanoma Families. Clin. Cancer Res..

[50] Lowenfels A.B., Maisonneuve P., DiMagno E.P., Elitsur Y., Gates L.K., Perrault J., Whitcomb D.C. (1997). Hereditary Pancreatitis and the Risk of Pancreatic Cancer. International Hereditary Pancreatitis Study Group. J. Natl. Cancer Inst..

[51] Lowenfels A.B., Maisonneuve P., Whitcomb D.C. (2000). Risk Factors for Cancer in Hereditary Pancreatitis. International Hereditary Pancreatitis Study Group. Med. Clin. N. Am..

[52] Rebours V., Boutron-Ruault M.-C., Schnee M., Férec C., Maire F., Hammel P., Ruszniewski P., Lévy P. (2008). Risk of Pancreatic Adenocarcinoma in Patients with Hereditary Pancreatitis: A National Exhaustive Series. Am. J. Gastroenterol..

[53] Oxford Centre for Evidence-Based Medicine: Levels of Evidence (March 2009). https://www.cebm.ox.ac.uk/resources/levels-of-evidence/oxford-centre-for-evidence-based-medicine-levels-of-evidence-march-2009.

[54] Lynch H.T., Lanspa S.J., Boman B.M., Smyrk T., Watson P., Lynch J.F., Lynch P.M., Cristofaro G., Bufo P., Tauro A.V. (1988). Hereditary Nonpolyposis Colorectal Cancer—Lynch Syndromes I and II. Gastroenterol. Clin. N. Am..

[55] Neumann H.P., Dinkel E., Brambs H., Wimmer B., Friedburg H., Volk B., Sigmund G., Riegler P., Haag K., Schollmeyer P. (1991). Pancreatic Lesions in the von Hippel-Lindau Syndrome. Gastroenterology.

[56] Lynch H.T., Richardson J.D., Amin M., Lynch J.F., Cavalieri R.J., Bronson E., Fusaro R.M. (1991). Variable Gastrointestinal and Urologic Cancers in a Lynch Syndrome II Kindred. Dis. Colon Rectum.

[57] Cox V.L., Saeed Bamashmos A.A., Foo W.C., Gupta S., Yedururi S., Garg N., Kang H.C. (2018). Lynch Syndrome: Genomics Update and Imaging Review. Radiographics.

[58] PLSD. https://plsd.eu/.

[59] Lucas A.L., Frado L.E., Hwang C., Kumar S., Khanna L.G., Levinson E.J., Chabot J.A., Chung W.K., Frucht H. (2014). BRCA1andBRCA2germline Mutations Are Frequently Demonstrated in Both High-Risk Pancreatic Cancer Screening and Pancreatic Cancer Cohorts: BRCA1/2Germline Mutations in Pancreatic Cancer. Cancer.

[60] Tulinius H., Olafsdottir G.H., Sigvaldason H., Tryggvadottir L., Bjarnadottir K. (1994). Neoplastic Diseases in Families of Breast Cancer Patients. J. Med. Genet..

[61] Greer J.B., Lynch H.T., Brand R.E. (2009). Hereditary Pancreatic Cancer: A Clinical Perspective. Best Pract. Res. Clin. Gastroenterol..

[62] Chappuis P.O., Ghadirian P., Foulkes W.D. (2001). The Role of Genetic Factors in the Etiology of Pancreatic Adenocarcinoma: An Update. Cancer Invest..

[63] Brentnall T.A. (2000). Cancer Surveillance of Patients from Familial Pancreatic Cancer Kindreds. Med. Clin. N. Am..

[64] Rutherford C.R., Rives T.A., Piecoro D.W., Dietrich C.S. (2024). Malignant STK11 Adnexal Tumor Harboring a Somatic Mutation in a Woman Previously Diagnosed with Mesothelioma, a Case Report. Gynecol. Oncol. Rep..

[65] Schneider K., Zelley K., Nichols K.E., Schwartz Levine A., Garber J. (1993). Li-Fraumeni Syndrome. GeneReviews(^®^).

[66] Dinarvand P., Davaro E.P., Doan J.V., Ising M.E., Evans N.R., Phillips N.J., Lai J., Guzman M.A. (2019). Familial Adenomatous Polyposis Syndrome: An Update and Review of Extraintestinal Manifestations. Arch. Pathol. Lab. Med..

[67] Pauli R.M., Pauli M.E., Hall J.G. (1980). Gardner Syndrome and Periampullary Malignancy. Am. J. Med. Genet..

[68] Stevenson J.K., Reid B.J. (1986). Unfamiliar Aspects of Familial Polyposis Coli. Am. J. Surg..

[69] Danesino C., Biglioli F., Moneghini L., Valli R., Olivieri C., Testa B., Baldo C., Malacarne M., Guala A. (2024). Pleomorphic Parotid Adenoma in a Child Affected with Cri Du Chat Syndrome: Clinical, Cytogenetic, and Molecular Analysis. Int. J. Mol. Sci..

[70] Leung R.S., Biswas S.V., Duncan M., Rankin S. (2008). Imaging Features of von Hippel-Lindau Disease. Radiographics.

[71] Elli L., Buscarini E., Portugalli V., Reduzzi L., Reduzzi C., Brambilla G., Menozzi F., Bardella M.T., Piodi L.P., Caldato M. (2006). Pancreatic Involvement in von Hippel-Lindau Disease: Report of Two Cases and Review of the Literature. Am. J. Gastroenterol..

[72] Graziani R., Mautone S., Vigo M., Manfredi R., Opocher G., Falconi M. (2014). Spectrum of Magnetic Resonance Imaging Findings in Pancreatic and Other Abdominal Manifestations of Von Hippel-Lindau Disease in a Series of 23 Patients: A Pictorial Review. JOP.

[73] Greenhalf W., Grocock C., Harcus M., Neoptolemos J. (2009). Screening of High-Risk Families for Pancreatic Cancer. Pancreatology.

[74] Grover S., Syngal S. (2010). Hereditary Pancreatic Cancer. Gastroenterology.

[75] LaFemina J., Roberts P.A., Hung Y.P., Gusella J.F., Sahani D., Fernández-del Castillo C., Warshaw A.L., Thayer S.P. (2009). Identification of a Novel Kindred with Familial Pancreatitis and Pancreatic Cancer. Pancreatology.

[76] Hruban R.H., Canto M.I., Yeo C.J. (2001). Prevention of Pancreatic Cancer and Strategies for Management of Familial Pancreatic Cancer. Dig. Dis..

[77] Lynch H.T., Brand R.E., Deters C.A., Shaw T.G., Lynch J.F. (2001). Hereditary Pancreatic Cancer. Pancreatology.

[78] Fernández E., Vecchia C., D’avanzo B., Negri E., Franceschi S. (1994). Family History and the Risk of Liver, Gallbladder, and Pancreatic Cancer. Cancer Epidemiol. Biomarkers Prev..

[79] Schenk M., Schwartz A.G., O’Neal E., Kinnard M., Greenson J.K., Fryzek J.P., Ying G.S., Garabrant D.H. (2001). Familial Risk of Pancreatic Cancer. J. Natl. Cancer Inst..

[80] Hassan M.M., Bondy M.L., Wolff R.A., Abbruzzese J.L., Vauthey J.-N., Pisters P.W., Evans D.B., Khan R., Chou T.-H., Lenzi R. (2007). Risk Factors for Pancreatic Cancer: Case-Control Study. Am. J. Gastroenterol..

[81] Shi C., Hruban R.H., Klein A.P. (2009). Familial Pancreatic Cancer. Arch. Pathol. Lab. Med..

[82] Kim M.P., Evans D.B., Vu T.M., Fleming J.B. (2009). The Recognition and Surgical Management of Heritable Lesions of the Pancreas. Surg. Oncol. Clin. N. Am..

[83] Jacobs E.J., Chanock S.J., Fuchs C.S., Lacroix A., McWilliams R.R., Steplowski E., Stolzenberg-Solomon R.Z., Arslan A.A., Bueno-de-Mesquita H.B., Gross M. (2010). Family History of Cancer and Risk of Pancreatic Cancer: A Pooled Analysis from the Pancreatic Cancer Cohort Consortium (PanScan). Int. J. Cancer.

[84] Eckerle Mize D., Bishop M., Resse E., Sluzevich J. (2009). Familial Atypical Multiple Mole Melanoma Syndrome. Cancer Syndromes.

[85] Hruban R.H., Canto M.I., Goggins M., Schulick R., Klein A.P. (2010). Update on Familial Pancreatic Cancer. Adv. Surg..

[86] Katabathina V.S., Buddha S., Rajebi H., Shah J.N., Morani A.C., Lubner M.G., Dasyam A., Nazarullah A., Menias C.O., Prasad S.R. (2021). Pancreas in Hereditary Syndromes: Cross-Sectional Imaging Spectrum. Radiographics.

[87] Welinsky S., Lucas A.L. (2017). Familial Pancreatic Cancer and the Future of Directed Screening. Gut Liver.

[88] Yoshida R. (2021). Hereditary Breast and Ovarian Cancer (HBOC): Review of Its Molecular Characteristics, Screening, Treatment, and Prognosis. Breast Cancer.

[89] Ngamruengphong S., Canto M.I. (2016). Screening for Pancreatic Cancer. Surg. Clin. N. Am..

[90] Llach J., Carballal S., Moreira L. (2020). Familial Pancreatic Cancer: Current Perspectives. Cancer Manag. Res..

[91] Giardiello F.M., Offerhaus G.J., Lee D.H., Krush A.J., Tersmette A.C., Booker S.V., Kelley N.C., Hamilton S.R. (1993). Increased Risk of Thyroid and Pancreatic Carcinoma in Familial Adenomatous Polyposis. Gut.

[92] Canto M.I., Harinck F., Hruban R.H., Offerhaus G.J., Poley J.-W., Kamel I., Nio Y., Schulick R.S., Bassi C., Kluijt I. (2013). International Cancer of the Pancreas Screening (CAPS) Consortium Summit on the Management of Patients with Increased Risk for Familial Pancreatic Cancer. Gut.

[93] Conroy T., Pfeiffer P., Vilgrain V., Lamarca A., Seufferlein T., O’Reilly E.M., Hackert T., Golan T., Prager G., Haustermans K. (2023). Pancreatic Cancer: ESMO Clinical Practice Guideline for Diagnosis, Treatment and Follow-Up. Ann. Oncol..

